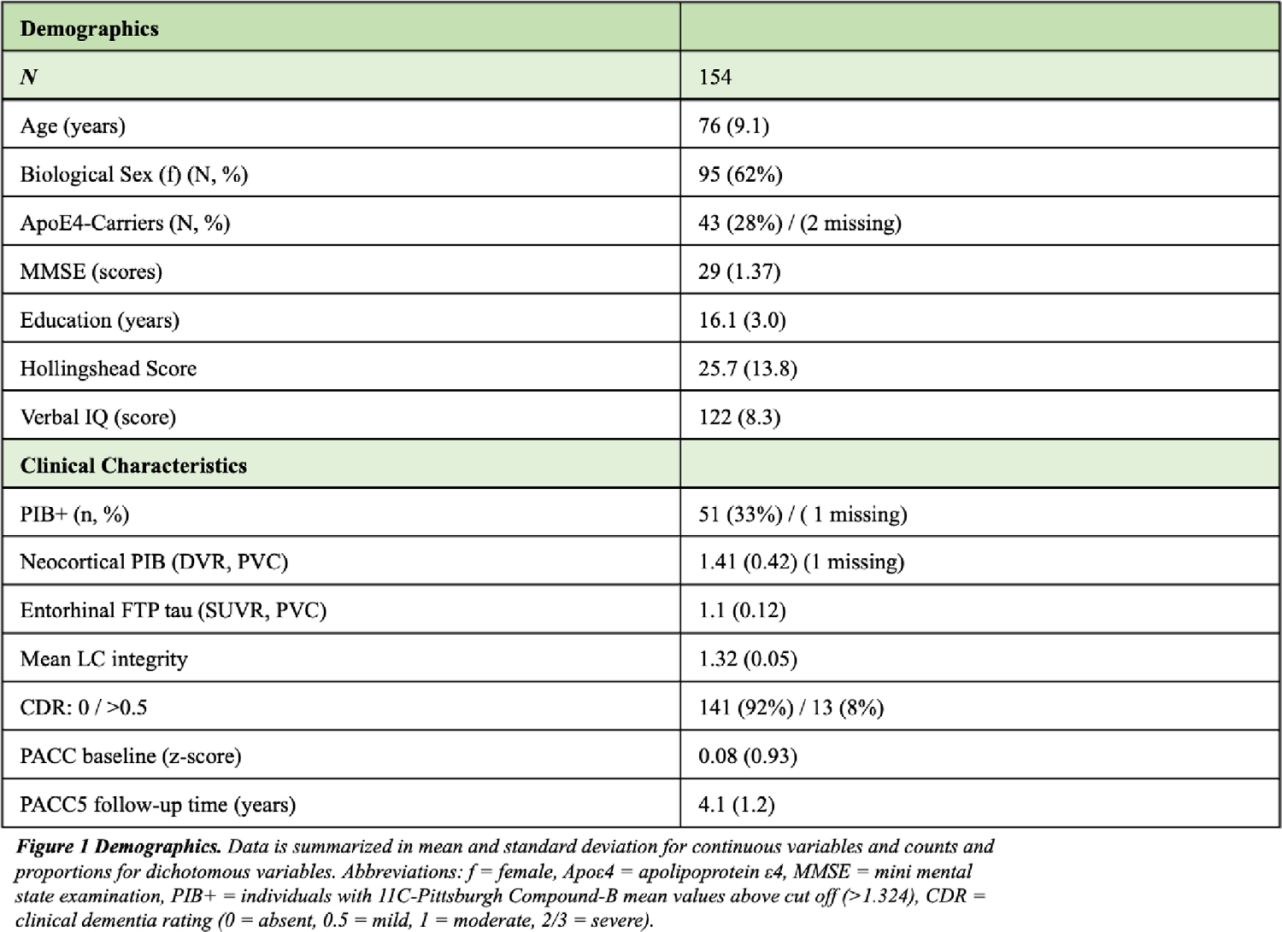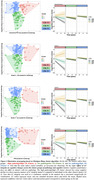# Higher locus coeruleus integrity and cognitive reserve attenuate tau‐related cognitive decline in older adults

**DOI:** 10.1002/alz.095547

**Published:** 2025-01-09

**Authors:** Lukas Heinrich, Joost M. Riphagen, Elouise A. Koops, Kathryn V Papp, Dorene M Rentz, Reisa A Sperling, Keith A Johnson, Heidi I.L. Jacobs

**Affiliations:** ^1^ Athinoula A. Martinos Center, Massachusetts General Hospital, Boston, MA USA; ^2^ Faculty of Psychology and Neuroscience, Maastricht University, Maastricht, Limburg Netherlands; ^3^ Massachusetts General Hospital, Harvard Medical School, Department of Neurology, Boston, MA USA; ^4^ Center for Alzheimer’s Research and Treatment, Brigham and Women’s Hospital, Massachusetts General Hospital, Harvard Medical School, Boston, MA USA; ^5^ Gordon Center for Medical Imaging, Massachusetts General Hospital, Harvard Medical School, Boston, MA USA; ^6^ Faculty of Health, Medicine and Life Sciences, School for Mental Health and Neuroscience, Alzheimer Centre Limburg, Maastricht University, Maastricht, Limburg Netherlands

## Abstract

**Background:**

Previous research suggests that locus coeruleus (LC) integrity may partially underlie cognitive reserve (CR), as demonstrated by positive associations between composite measures of CR and MRI‐based integrity of the LC, one of the first regions in the brain to accumulate tau pathology. We investigated whether higher MRI‐based integrity of the LC confers resilience to cognitive decline related to Alzheimer’s disease pathology in individuals with higher cognitive reserve.

**Method:**

Individuals (n = 154) with LC‐MRI, 18F‐Flortaucipir (FTP‐tau), and 11C‐Pittsburgh Compound‐B (PIB‐amyloid) PET were selected from the Harvard Aging Brain Study (Figure 1). Cognition over time was modeled using the PACC5 composite score. CR was quantified using a composite of verbal IQ, years of education, and occupational attainment (i.e., Hollingshead score). We used a data‐driven approach to cluster participants on the basis of both their CR and AD pathology. K‐means clustering was performed to group individuals based on CR and entorhinal FTP, FTP‐based Braak stage III or IV composites, or neocortical PiB. The relationship between LC MRI‐integrity and cognition over time across the cluster‐derived pathology‐cognitive reserve groups was assessed using linear mixed effects modeling (adjusted for age and sex).

**Result:**

Cluster analysis consistently identified three groups (Figure 2, left side): High pathology/high CR; low pathology/high CR, and low pathology/low CR. Throughout all analyses, the high pathology/high CR cluster showed less steep cognitive decline over time in a dose‐response manner of LC integrity (Figure 3, right side). Attenuated cognitive decline was observed at higher levels of LC integrity in individuals with high cognitive reserve and elevated levels of FTP in entorhinal cortex compared to low FTP‐tau/low CR (*ß* = ‐6.19, *p*<0.001) and low FTP‐tau/high CR (*ß* = ‐6.72, *p*<0.001). Similar group comparisons were observed after grouping for tau in Braak stage 3 (*ß* = ‐10.78, *p*<0.001; *ß* = ‐10.37, *p*<0.001) and Braak stage 4 brain areas (*ß* = ‐9.61, *p*<0.001; *ß* = ‐8.80, *p*<0.001) and with PIB DVR (*ß* = ‐8.82, *p*<0.001; *ß* = ‐8.03, *p*<0.001).

**Conclusion:**

These findings reveal that higher LC integrity may constitute a brain reserve measure bolstering CR in conferring resilience against cognitive decline in the face of elevated AD‐pathology. Future research aims to extend this research to LC function and networks.